# Using choice modelling to inform service sustainability for dementia Meeting Centres for people living with dementia in the UK

**DOI:** 10.1080/13607863.2024.2375609

**Published:** 2024-07-09

**Authors:** Michela Tinelli, Thomas Morton, Jennifer Bray, Catherine Henderson, Faith Frost, Shirley Evans

**Affiliations:** a Care Policy Evaluation Centre (CPEC), the London School of Economics and Political Science, Houghton St, London, UK; b The Association for Dementia Studies, St Johns Campus, University of Worcester, Worcester, UK

**Keywords:** Dementia, meeting centre, discrete choice experiments, choice modelling, preferences, carers

## Abstract

**Objectives:**

This study explores the preferences and willingness-to-pay (WTP) of carers for Meeting Centres (MCs) attributes in assisting individuals with mild to moderate dementia.

**Method:**

Preferences from 108 carers, gathered through UK-wide MC networks, were collected using a Discrete Choice Experiment survey. The survey incorporated attributes derived from evidence synthesis and lay consultation. A regression model estimated preference weights and marginal WTP for a change in attributes one a time within the MC support ‘package.'

**Results:**

Carers preferred MCs offering a balanced mix of practical activities and emotional support, along with flexibility without booking requirements and low costs. Social opportunities and the frequency of the meeting were not prioritised. Respondents expressed a WTP of £43 to stay with ‘My MC,’ the preferred option, compared to transitioning to an alternative in-person MC, all else being equal. Various factors, including attendance modality, the relationship with the supported person, age, and gender, influenced carers’ choices.

**Conclusion:**

These findings offer valuable insights into carers’ preferences, priorities, and WTP within MC support for those with mild to moderate dementia. Understanding these factors can guide the implementation and sustainability of MCs, ensuring alignment with carers’ needs and preferences and, ultimately, enhancing support for individuals with dementia.

## Background

The goal of improving support for people with mild to moderate dementia to live at home in their communities is a global public health objective (Alzheimer’s Disease International, 2022; World Health Organisation, 2017). However, community-based interventions in various parts of the UK may not be adequately equipped to meet the needs of this growing population, and the COVID-19 pandemic has further intensified the pressure on these services (Care Quality Commission, 2022).

In this context, community-led interventions can play a vital role in supporting individuals with dementia post-diagnosis (Brooker et al., 2018; Evans et al., 2020; Lord et al., 2020) and delaying the need for residential care (Dröes et al., 2004a, 2006).

Meeting Centres (MCs) for people living with dementia have been widely adopted in the UK since 2015. There are seven such centers across the country. MCs build on a successful Dutch model (Dröes et al., 2003; Dröes et al., 2004a, 2004b) that differs from traditional day care in being aimed at people with mild to moderate dementia, supporting not only to people with dementia but also their families, friends, and other carers. They aim to connect individuals with dementia to their community and create a supportive social environment. They decrease behavioural issues and improve mood, self-esteem and wellbeing in individuals with dementia, while enhancing caregivers’ sense of competence and ability to cope and having a positive impact upon their mental health (Dröes et al., 2004a, 2004b, 2006; Brooker et al., 2018; Evans et al., 2020).

MCs align with the principles of dementia friendly communities and offer a higher level of support compared to dementia cafes, which are generally run for two-hour periods once or twice a month. The core of an MC is a small social club, typically accommodating around 10–15 people per day, along with their supporting family members, friends, and carers. These clubs are in ordinary community buildings, conveniently accessible to where people live. They usually operate up to three days a week, providing opportunities for building friendships, receiving peer support, understanding dementia-related challenges, accessing help by acting as a hub for health and social care professionals and by signposting and referring, and preparing for the future.

In addition to social support, MCs also offer evidence-based post-diagnostic interventions tailored to the needs of their members. These interventions are facilitated by a small team of staff and volunteers who receive training in person-centred dementia care and the Adjusting to Change Model (Brooker et al., 2017). The aim is to provide personalised care and support that helps people living with dementia adapt to their changing circumstances and cope with the challenges they face.

The appeal of MCs lies in their community-based nature, which fosters a sense of belonging and provides opportunities for individuals with dementia and their carers to engage in meaningful activities and socialise with others facing similar challenges. The person-centred approach of MCs aims to address the individual needs, preferences, and aspirations of people with dementia, promoting their overall well-being and quality of life.

While MCs have shown promise, sustaining them beyond the initial one- or two-year period has proven challenging for various reasons, including funding constraints, limited resources, and difficulties in maintaining volunteer engagement. Addressing these challenges is crucial to ensure the long-term viability and impact of MCs and similar community-led interventions.

As the range of provision an MC can offer is multifaceted, with various potential benefits to attendees, it is important to understand which elements people most value to maximise the appeal of MCs to its target population and, by extension, its chances of sustained operation (Orellana et al., 2023). As part of a larger realist evaluation programme that aimed to understand the factors affecting the sustainability of existing MCs, Discrete Choice Experiments (DCEs, see below) were used to explore what people across the UK value and are willing to pay for MCs.

## Methods

Choice modelling is a survey-based method that captures individual decision-makers’ preferences regarding different scenarios or service provisions using DCE questionnaires. Each alternative scenario is described by multiple attributes or characteristics, and participants are asked to make choices between two or more competing scenarios. These choices help determine how preferences are influenced by each attribute and their relative importance (Tinelli, 2016). Examples of their application to measure the value of services for people with dementia collecting preferences from the general public, the neurologist, the carers as well as the individuals with dementia are provided elsewhere (Dranitsaris et al., 2023; Speckemeier 2023; Teahan et al., 2021; Wammes et al., 2023).

When analysed, DCE data allows for the measurement of the overall value attached to different alternatives and helps identify optimal service provisions that meet stakeholder requirements and have the best chance of long-term sustainability. By including a cost attribute, choice modelling also enables the weighing of benefits and costs associated with service provisions. It can provide insights into stakeholders’ willingness to pay for a particular service provision and how their preferences and willingness to pay may vary between current options and their preferred alternatives.

### Selection of DCE items and DCE choice sets

In this study, the initial choice of DCE characteristics was informed by previous research on the essential features of MCs (Evans et al., 2022) and the Adjusting to Change Model (Brooker et al., 2017) and by a realist review on the sustainability of community-based groups and activities for people living with dementia (Morton et al., 2022).

For the experiment, the MC characteristics, or ‘attributes’, each featured a range of options, or ‘levels’. These attributes and levels were validated and refined in with MC members (see Population section). Four MC leads across the three case study sites also provided data on the frequency of attendance and modalities and the range of possible levels for the cost attributes.

**Figure 1.**
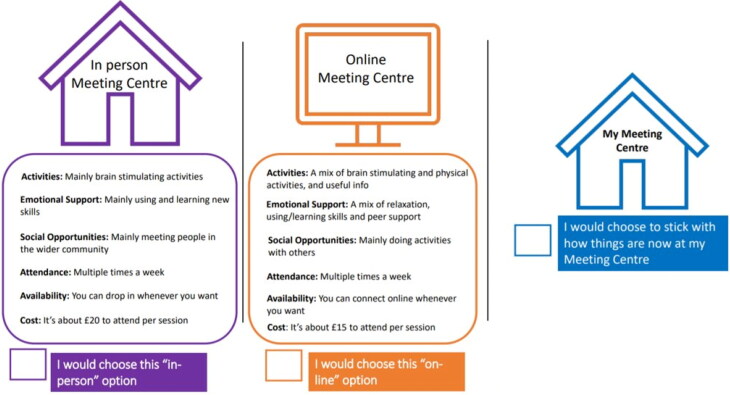
Example of choice set.

The DCE was conducted following the steps outlined in the standard guidelines (Reed Johnson et al., 2013). In our experiments, participants were presented with three scenarios (‘In person MC’, ‘Online MC’ and ‘My MC’), each with the same attributes, but varying levels of each attribute. For example, the attribute ‘out of pocket price’ could have levels of ‘no cost’, ‘£5’, ‘£10’, ‘£15’, ‘£20’, and ‘£25 or more’. ‘Practical activities and support’ could vary between ‘information and signposting’, ‘physical fitness and well-being activities’, ‘brain stimulating activities’, or ‘equal mix of the above’ The full list of attributes, levels, and definitions pertaining to the current study are presented in Figure 1 and Tables 1 and 2.

**Table 1.** Descriptive characteristics about the carer and the person they care for collected from the respondents who returned a completed DCE choice set.

	About the carer % (*n* = 108)	About the person they care for % (*n* = 108)
**Gender**		
Male	23.53	42.16
Female	75.49	56.86
Other	0	0.00
Prefer not to say	0.98	0.98
**Age**		
Mean	66.42	81.91
SD	15.37	7.66
**Ethnicity**		
White	99.02	98.02
Black	0	0
Mixed ethnic group	0	1.98
Asian	0	0
Other	0	0
Prefer not to say	0.98	0
**Relationship to the person they support**		
Spouse or partner	48.51	n/a
Son or daughter	38.61	n/a
Other family member	3.96	n/a
Friend	3.96	n/a
Other	4.95	n/a
**Attendance**		
In person	100	n/a
On line	0	n/a
**Attendance, year**		
Mean	2.29	n/a
SD	1.46	n/a
**Attendance with:**		
I usually attend the Meeting Centre together with the person I support	50.00	n/a
I attend the Meeting Centre but the person I support is no longer able to attend	3.92	n/a
I attend the Meeting Centre as a former carer	0.98	n/a
I do not usually stay to attend the Meeting Centre myself	45.10	n/a
**Health status**		
Very good	n/a	9.09
Good	n/a	18.18
Average	n/a	44.44
Poor	n/a	24.24
Very poor	n/a	4.04

**Table 2.** Regression modelling and marginal WTP estimates.

	β number^a^	β value	SE^b^	P.val	Marginal WTP (£)	SE^b^
**Practical activities and support**						
Mainly information and signposting, such as carers’ talks and visits from health professionals	β1	−1.60	1.65	0.33	n/a	n/a
Mainly physical fitness and well-being activities, such as walking, exercise or dancing	β2	−0.92	0.61	0.10	−21.38	0.25
Mainly brain stimulating activities, such as quizzes, discussions, art, music and games	β3	−0.45	0.48	0.10	−10.41	0.12
(Compared to an equal mix of the below)						
**Emotional support**						
Mainly an opportunity for people to talk with others, give and receive support (peer support)	β4	−1.42	0.50	0.01	−32.84	0.38
Mainly a chance for people to use their existing skills and learn new ones	β5	2.34	1.08	0.03	54.34	0.63
Mainly a relaxing atmosphere	β6	−0.95	0.88	0.28	n/a	n/a
(Compared to an equal mix of the below)						
**Social opportunities**						
Mainly a chance for people to do activities with others	β7	−0.82	0.73	0.26	n/a	n/a
Mainly a chance to meet other people in the wider community (including trips out or visitors to the Meeting Centre)	β8	−0.67	0.92	0.46	n/a	n/a
Mainly a chance for people to chat with friends at the Meeting Centre	β9	−0.05	0.66	0.94	n/a	n/a
(Compared to an equal mix of the below)						
**How often**						
Multiple times a week	β10	0.29	0.46	0.52	n/a	n/a
(Compared to once a week at most)						
**How available is it**						
You can drop in or connect online on a range of days	β11	−0.83	0.73	0.26	n/a	n/a
You can drop or connect online on a specific day	β12	−0.54	0.62	0.39	n/a	n/a
You have to book a pre-appointed time	β13	−0.85	0.52	0.10	−19.67	0.23
(Compared to you can drop in or connect online whenever you want to (and don’t have to book)						
**Cost**						
Cost per visit (£)∼ (Out of pocket money paid by the carer)	β14	−0.04	0.01	0.10	n/a	n/a
	ASC name	ASC value	SE	P.val	Marginal WTP	SD
**Alternative** **Meeting Centre **						
(Compared to my Meeting Centre)						
In-person Meeting Centre	ASC _In-person_	−1.86	1.00	0.06	−43.17	0.50
Online Meeting Centre	ASC _online_	−3.78	2.15	0.36	n/a	n/a

^a^Regression coefficients and variable names (see full model in Appendix 1).

^b^SE = standard error. ∼The levels used in the design were: It’s free, about £5, about £10, about £15, about £20, about £25. Note: We calculated WTP only for those β coefficients that reached statistical significance at the 10% level.

The experimental design we considered to build the DCE questions had three named scenarios, one attribute with two levels, four attributes with four levels and one attribute with six levels, resulting in 192 potential choice sets (2^1^ × 4^4^ × 6^1^). A design maximised for the multinomial logit (MNL) model based on D-efficiency criteria was used to generate 12 choice sets using the design software Ngene (https://www.choice-metrics.com/). An example of one of the 12 choice sets is shown in Figure 1. We examined the scenarios for any illogical combination and found that all attributes and their respective levels were applicable and coherent across all scenarios.

For individuals with mild cognitive impairment, it has been found that five to six choice sets are more optimal (Milte et al., 2014). Therefore, to accommodate these considerations, the full design of the survey was divided into three versions, with four specific choice sets per version plus one consistency test question for a total of five questions each. A dominant choice set (in which one service contains all preferred attribute levels and the other contains the least preferable levels) was added to check for consistency (Ryan et al. 2005; see Appendix 1).

An experimental design technique was implemented to ensure a balanced representation of attribute levels across the four versions of the DCE questionnaire. This approach aimed to optimise participant engagement and comprehension while maintaining the integrity of the survey design. Reed Johnson et al. (2013) provides additional information on the use of a blocking design to achieve attribute level balance.

### Preparatory work with patient, public and practitioner involvement and engagement (PPPIE) consultations

In the summer 2021, we interviewed nine people (six people living with dementia, two family carers and one MC volunteer) asking people living with dementia and family carers generally about the nature of the support they received and their values regarding MC, from which attributes and levels were drawn. Following that, the initial version of the DCE survey was presented to a separate group ten stakeholders (seven in an MC management/governance role; two family carers; one person living with dementia) for their feedback on the presentation, wording and format of the survey, and views on the acceptability and feasibility of administering the DCE survey to both carers and people with dementia. Based on their feedback, we incorporated ‘Virtual MCs’ as a distinct option. Additionally, the questionnaire was tailored to focus specifically on carers, allowing them to represent both themselves and the individuals they care for as a dyad.

In October 2021, an internal pilot study with individuals who provided support or care to someone with dementia aimed to further consolidate comprehension and understanding of the choice set tasks, attributes and their levels as well as the functioning of the DCE survey. Results from the analysis of the pilot data were used to test the face validity of the survey instrument. The pilot study included responses from the first 17 participants (nine *via* email and eight *via* paper copy) from one MC case study site. We tested data collection and cleaning processes as well as the econometric model with the analysis of 13 questionnaires. The pilot phase revealed no procedural issues with the experimental design of the survey. Consequently, no additional revisions were deemed necessary, and the data collected during this phase were utilised within the main analysis.

### The DCE survey

The main experiment was conducted between January and October 2022 with individuals who provided support or care to someone with dementia, enabling them to report preferences on behalf of the attendees. Those individuals who, after receiving information about the study, provided consent, subsequently participated in the survey. The surveys were handed out in paper form by both researchers and staff members. Additionally, they were made available online through email promotions and social media channels across all UK MCs. Participants at each MC were randomly given one of the three DCE questionnaires to complete. The first module of the questionnaire asked about their current MC and presented the DCE choice sets. The second module collected information collect basic demographic data about the carer (ie: age, gender, ethnicity, relationship to the person they support, attendance – whether in person/online, who they accompany and duration), and about the person they care for (ie: age, gender, ethnicity and health status).

### Sample size

According to the current theory of sampling, determining the appropriate sample size is influenced by various factors related to the study design, including the number of attributes, the size of the population, and the desired statistical power of the model derived. Considering suggestions in the literature and previous studies utilising DCE methodology (Ali and Ronaldson 2012; Chester et al., 2018; Johnson and Orme 2010; Netten et al., 2012; O’Philbin et al., 2020), an initial recruitment target of 250 participants was set for this study to allow for two groups to be considered and compared, an individual living with dementia (125) and their carers (125). However, based on the PPPIE feedback (see PPPIE above) to focus the survey on carers, we reduced our original target sample size to 125 people.

### Data analysis

DCE data were analysed applying regression modelling (see Appendix 1). Subgroup analysis was conducted to investigate how various variables, including gender, age, and health status, influenced people’s preferences. Difference between groups was tested in WTP estimates was investigated with interacting terms in regression modeling (see Appendix 1). Data were managed in Microsoft Excel (cleaning and organising), SPSS version 24 (descriptive data) and Nlogit version 6 (DCE data).

## Results

### Choice modelling and DCE survey

#### DCE survey responses and constant preference for DCE alternatives

A total of 122 questionnaires were returned, representing 98% of the target sample size (122 out of 125). This accounts for approximately 30% of the total number of carers connected with MCs across the UK at that time, which was 389. About 50% of questionnaires were returned online with the remaining being paper copies collected at the MCs. We collected comparable numbers of questionnaires with different DCE choice versions (versions 1: 36%, version 2: 29%, version 3: 35%). 108 DCE questionnaires classed as being complete (89% of 122) and all 108 of these passed the consistency test (Ryan et al. 2005; see Appendix 1) and demonstrated a consistent response pattern throughout the questionnaire. The respondents were predominantly white female, 66 years old and taking care of a family member (either spouse or parent; Table 1).

About 70 people (65%; 70/108) reported constant preferences for ‘My MC’ (mainly characterised by an equal mix of practical activities and support, an equal mix of emotional support, an equal mix of social opportunities, available one a week at most, cost per visit of £10 or more—see Figure 2).

Figure 2.What the Meeting Centre currently offers. Attribute 1: Practical activities and support. Attribute 2: Emotional support. Attribute 3: Social opportunities. Attribute 4: Availability. Attribute 5: Cost per visit (£).

**Figure:**
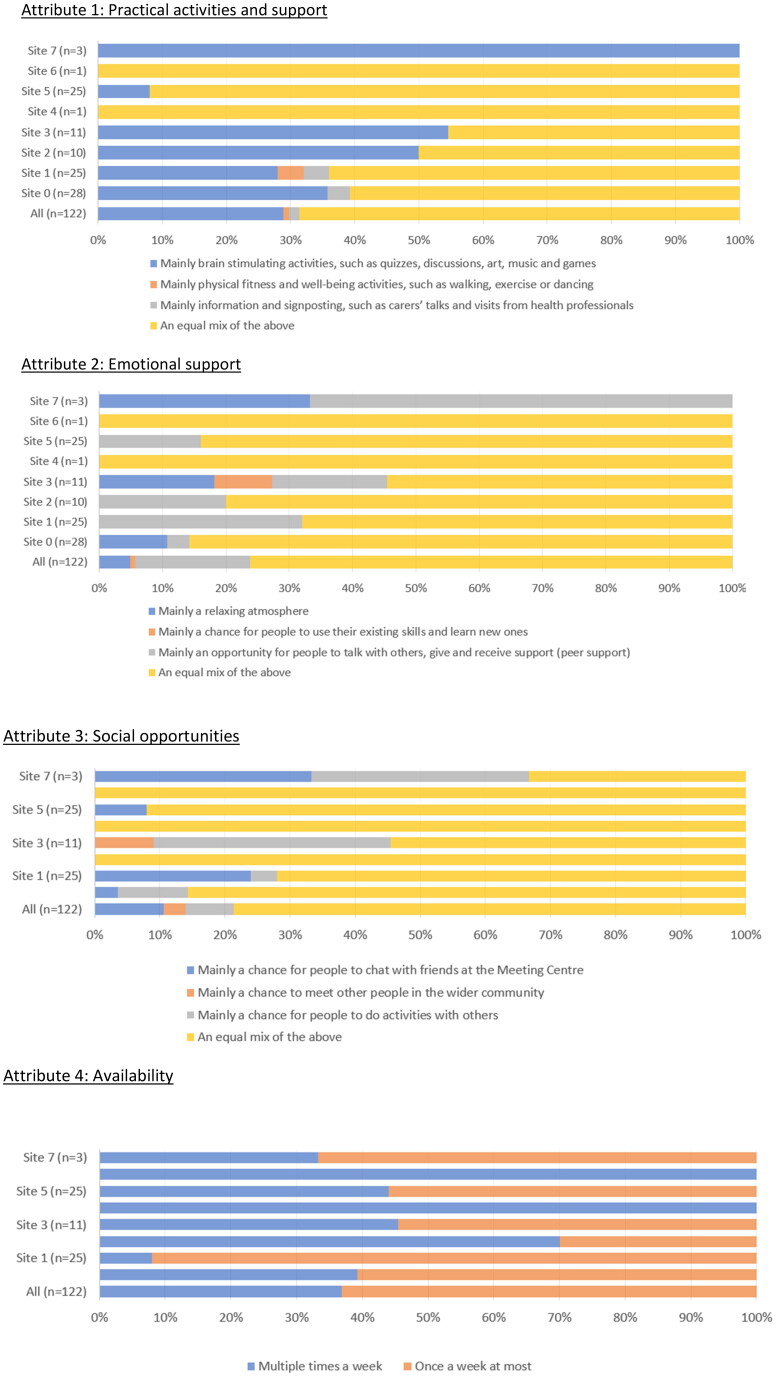

**Figure:**
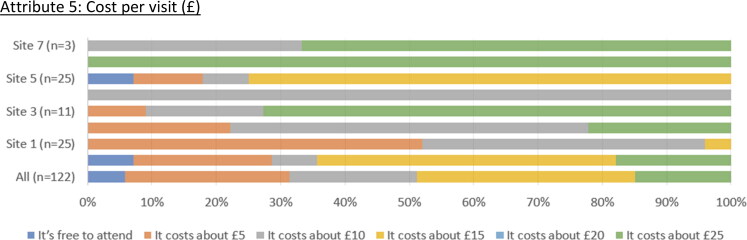

#### Analysis of the DCE data

##### Preference results (Table 2)

The regression results showed that, in terms of emotional support, respondents showed a preference for primarily using their existing skills and learning new ones, as opposed to opting for an equal mix of various types of support. Th negative sign of the cost attribute (β14 = −0.04) indicated that the smaller the cost is, the greater the likelihood of choosing the MC. Overall, the signs of the coefficients aligned with our expectations (details in Appendix 1), except for (β4 = −1.42). Contrary to our anticipation of a positive value, it turned out negative, indicating that people valued a mix of emotional support more than peer support.

In this context, respondents did not find Online MCs valuable (p_ASConline_ = 0.36). However, they showed a preference for ‘My MC’ over the ‘in-person MCs’ alternative, with a *p*value of 0.06, indicating statistical significance at 10%. While all service characteristics are appreciated, there is no prioritisation for any type of social opportunities (p_β7_ = 0.26, p_β8_ = 0.46, and p_β9_ = 0.94) or the frequency of the meetings (p_β10_ = 0.52), being able to drop in or connect online on various days (p_β11_ = 0.26), or on a specific day (p_β12_ = 0.39).

The preferred characteristics, ranked from most to least preferred based on the size of the coefficients, are: utilising existing skills and acquiring new ones, a balanced combination of practical activity and support, a balanced mix of emotional support, no requirement for booking, and lower costs.

##### Ranking different MC configurations (Table 3)

We calculated the overall satisfaction for various MC configurations, each with different combinations of features. Then, we ranked these configurations from the most preferred to the least preferred. Higher utility scores indicate greater preference. The top five MC configurations are presented in Table 3. The most preferred MC was characterised as offering:

**Table 3.** Ranking for the top 5 MCs.

	Each MC option is defined:						
	Practical activities and support	Emotional support	Social opportunities	How often can you attend?	How available is it?	Cost per visit	Score^a^
**MC Option 1 (most preferred):** (Ranked first)	A mix of brain-stimulating and physical activities, and useful info	Mainly using and learning skills	A mix of chatting with friends, meeting new people and doing activities	Multiple times a week	You can go whenever you want	It’s free	2.64
**MC Option 2:** (Ranked second)	A mix of brain-stimulating and physical activities, and useful info	Mainly using and learning skills	Mainly chatting with friends	Multiple times a week	You can go whenever you want	It’s free	2.59
**MC Option 3:** (Ranked third)	A mix of brain-stimulating and physical activities, and useful info	Mainly using and learning skills	A mix of chatting with friends, meeting new people and doing activities	Multiple times a week	You can go whenever you want	About £5	2.42
**MC Option 4:** (Ranked fourth)	A mix of brain-stimulating and physical activities, and useful info	Mainly using and learning skills	A mix of chatting with friends, meeting new people and doing activities	No more than once a week	You can go whenever you want	It’s free	2.34
**MC Option 5:** (Ranked fifth)	A mix of brain-stimulating and physical activities, and useful info	Mainly using and learning skills	A mix of chatting with friends, meeting new people and doing activities	Multiple times a week	You can go whenever you want	About £10	2.21

^a^The utility score attached to each option (the higher the score, the more preferred it is).

- A mix of brain-stimulating and physical activities and useful information;
- The chance to use and learn skills as a means of emotional support;
- a mix of social opportunities (chatting with friends, meeting new people and doing activities);
- Being able to attend multiple times a week;
- Having the opportunity to attend whenever the person wants;
- Free to attend.

The most common MCs currently available presented similar characteristics to the most preferred MC configuration as reported in Appendix 2.

##### Willingness to pay (Table 2)

Respondents were willing to pay an extra £43 to stay with ‘My MC’ compared with shifting to an alternative in person MC (everything else equal, Table 2). WTP is a measure of the perceived value or preference placed on the choice between ‘My MC’ and an alternative in person MC and it does not imply an actual additional payment of £43.

For example, when looking at the WTP for a change for one individual attribute, people were willing to pay an additional £33 for a change in emotional support. This means they valued a mix of relaxation, using/learning skills, and peer support over mainly having an opportunity to talk with others and give/receive support.

##### Explicative factors for WTP (Appendix 3)

Our subgroup analysis indicates that among carers who attend the MC, those attending alongside the person they support expressed a lower WTP for ‘My MC’ compared to other carers. People who had formerly been carers preferred transitioning from ‘My MC’ to an alternative ‘in-person MC’, unlike their counterparts who have a stronger preference for ‘My MC’. Younger carers exhibited a lower WTP for ‘My MC’ compared to older carers. On the other hand, male carers showed a higher WTP for ‘My MC’ compared to female carers.

Carers supporting their parents are willing to pay £15 for a shift from ‘My MC’ to an alternative in-person option. No notable differences in WTP estimates were found when considering other relationship types. For those caring for an older person, their WTP for ‘My MC’ is lower compared to those caring for a younger person. In cases where the person being cared for is male (typically a husband or father), there is a preference for shifting from ‘My MC’ to an alternative ‘in-person MC’, with a willingness to pay £18 for this change. WTP estimates do not show significant differences when considering the health status of the person being supported. For more details see Appendix 3.

## Discussion

The DCE survey yielded positive results, achieving a response rate of 30% or more of the total population of carers approached through their MC, despite the challenges posed by the COVID-19 pandemic. Based on the responses received, carers expressed their preference for an ideal MC that incorporates a combination of brain-stimulating and physical activities, as well as the provision of useful information. Carers showed a preference for a mix of opportunities for socialising, both with existing friends and by meeting new people. They valued the ability to attend the MC multiple times per week and appreciated the flexibility in terms of attendance timing. The preferred option would be accessible free of cost. However, carers acknowledged the value of the service received and expressed willingness to pay for it, with overall satisfaction with the current costs incurred. Among the most common MCs currently available, the preferred option was associated with a cost-per-visit of £15.

The findings from the DCE survey targeted to carers of people with dementia were triangulated with three focus groups with MC members conducted as part of the larger study (Morton et al., 2022). From the focus groups, it emerged that some members did not highly value signposting and information. However, it’s important to note that MCs are also for carers and the DCE data showed they do find such resources important. One of the key roles of MCs is to serve as a hub, reducing the need for individuals to actively search for additional support. The primary motivation for attending MCs was to have fun, socialise, engage in activities, and get out of the house. While signposting and information may still be provided in some capacity, they were not the main driving factors for members to attend MCs. Relaxation was not considered a priority for members. Many members expressed being under-stimulated at home, and they sought out MCs specifically for activities and social interactions. The need for stimulation and engagement outweighed the desire for relaxation.

The social aspects of MCs, such as doing activities together, meeting new people, and meeting with friends, were not clearly differentiated by participants. It suggests that these social elements were interconnected and intertwined for members, and they did not perceive them as distinct and separate experiences. Opportunities for socialising were, however, consistently highly valued in both the triangulation focus groups and interviews conducted as part of the wider study (Morton et al., 2022), in contrast to the carer-completed DCE. This suggests socialising is less of a priority for carers than for members living with dementia, and possibly that carers can underestimate the importance of socialising and social inclusion for those that they care for (Shannon et al., 2018; Carr et al., 2022).

Surprisingly, peer emotional support was not explicitly valued in the DCE. However, in previous interviews conducted as part of the larger study (Morton et al., 2022), members spoke extensively about the importance of emotional support from peers. It is important to note that not all carers actively seek this support, and it may be something that occurs more organically or implicitly rather than being actively and consciously sought out.

These findings highlight the nuanced perspectives of MC members and emphasise the primary motivations and preferences that drive their participation. While some elements, such as signposting and relaxation, may not be highly valued, it is crucial to consider the context and the varying needs and expectations of individuals within the MC setting. Understanding these trends can inform the development and refinement of MC programmes to better align with the desires and priorities of members, ultimately enhancing their overall experience and well-being.

Also, the study revealed that carers’ choices were influenced by various factors related to the carer themselves. These factors included the relationship with the person they support, the preferred attendance modality, as well as age and gender. For example, respondents reported lower marginal WTP for the ‘My MC’ option when attending together with the person they support. This finding may be influenced by the fact that Welsh MCs, as well as many Scottish MCs, which had good response rates to the questionnaire, tend to have lower costs for members but higher carer attendance and engagement than England. Also, we may argue that there has been a shift in perceptions of what a MC entails. Originally, MCs were seen as places that catered to the needs of both members and family carers in a safe and enjoyable manner, where active support was provided to both individuals with dementia and their family carers (Brooker, 2020). However, the DCE findings suggest that MCs are now increasingly viewed as places that provide respite for family carers. Depending on this perspective, the expectations regarding costs and carer engagement may differ. If seen as a formal service, higher costs may be expected with lower carer engagement. On the other hand, if viewed as an informal social club, lower costs may be anticipated with higher carer engagement.

The study faced a few limitations that impacted recruitment, data collection, and the design of the questionnaire. Involving the original sample of people with dementia and their carers posed challenges due to various factors. For example, dementia-related cognitive decline may impede effective communication and participation in research, with individuals facing challenges in recalling past experiences and expressing preferences. Also, ethical concerns regarding informed consent and capacity pose further obstacles when involving those with dementia in research, making it particularly challenging to ensure full comprehension of research aims, procedures, and risks. By exclusively targeting carers, the pool of potential respondents was reduced, but the revised target sample size of carers was successfully reached. The level of engagement and involvement of carers with individual MCs varied significantly across locations. In areas with low carer engagement, there was a lack of response to requests for questionnaire completion.

The impact of the COVID-19 pandemic further exacerbated these recruitment challenges, for example lower attendance at MCs than usual due to social distancing rules and people being reluctant to leave the house. Many existing MCs had closed their doors and the opening of planned MCs was delayed. This resulted in a smaller number of MCs available for distribution and lower attendance, particularly with lower carer involvement. The COVID-19 pandemic also affected the engagement of local MC staff in distributing the DCE, as they faced challenges such as limited resources and ‘questionnaire fatigue’. Despite ongoing requests and instructions from the research team, some MCs were not actively involved in distributing the questionnaires. While efforts were made to physically guide individuals through the completion of paper copies, geographical constraints limited the reach of this approach.

The questionnaire results demonstrated a lack of interest in virtual options. The choice to incorporate the ‘Online MC’ option in the DCE design was influenced by the circumstances during the COVID-19 pandemic. As soon as in-person meetings, even outdoors once a week, became feasible, the online aspect lost significance. Additionally, there was the challenge that the online option was less accessible to, or not preferred by, individuals with more moderate dementia. Another factor was the uncertainty about the duration of the pandemic and social distancing measures. The results might have been different if the need for online options had persisted for a more extended period. Excluding this option from the questionnaire may have simplified the questionnaire design and potentially yielded more robust results. Nevertheless, it can be concluded that there is a clear preference for face-to-face meetings based on the practice and outcomes observed.

Regardless the study limitations, choice modelling confirmed to be a useful tool to inform decision making in social care for dementia (Chester et al., 2018; Dranitsaris et al., 2023; O’Philbin et al., 2020; Speckemeier 2023; Teahan et al., 2021; Wammes et al., 2023). The insights emerging from the analysis of DCE survey data can indeed enhance stakeholder engagement as a strategy for advancing implementation and understanding stakeholders’ WTP (Salloum et al., 2017). They can contribute to stakeholder engagement and implementation efforts in multiple ways.

In this study DCEs survey method allow carers to express their preferences by making choices between different scenarios or options. This approach provides a structured framework for stakeholders to articulate their priorities and trade-offs. By involving our PPPIE stakeholders in the design and implementation of DCEs, their engagement is heightened, and their perspectives are directly incorporated into the decision-making process.

DCEs survey method involve identifying and defining attributes or characteristics of the intervention or service under consideration. Engaging our PPPIE stakeholders in this attribute selection process ensured that the identified attributes are relevant, meaningful, and align with stakeholders’ values and preferences.

The DCE data presented here incorporate a monetary component that measures stakeholders’ WTP for different features of the MC service. This allows decision-makers to understand the economic value that stakeholders place on specific aspects of the intervention. By involving stakeholders in the assessment of WTP, it provides them with a direct influence on resource allocation decisions and can help align implementation efforts with stakeholders’ preferences.

The DCE approach provides a transparent and systematic approach to capturing stakeholder preferences. The results were presented and shared with stakeholders as part of a workshop discussion, promoting transparency in the decision-making process.

Choice modelling facilitates discussions about trade-offs among different features of MCs. Participant carers could evaluate the importance of each attribute and make choices based on their preferences. This allows for a more nuanced understanding of stakeholders’ preferences and can inform MC implementation and sustainability in a way that better aligns with stakeholder priorities.

Overall, the study findings shed light on the complex interplay between carer characteristics, preferences and personal experiences, when it comes to the cost and engagement expectations of MCs. Understanding these factors can help tailor and improve the implementation of MCs to better meet the diverse needs of carers and individuals with dementia.

## Conclusion

In summary, the outcomes derived from the choice modelling illuminate the varied preferences of carers within MCs, emphasising the necessity for customised programmes that address a range of activities and social interactions. Despite encountering obstacles, the study emphasises the importance of understanding stakeholder dynamics, providing valuable insights to improve the implementation and sustainability of MCs. In conclusion, these findings contribute to the ongoing research to tailor dementia care services to better align with the needs of carers and individuals with dementia throughout the UK.

## Ethical approval

Favourable ethical opinion was received from the Health Research Authority research ethics committee (Wales REC4 21/WA/0185).

## Disclaimer

The views expressed are those of the authors and not necessarily those of the NIHR or the Department of Health and Social Care.

## Appendices

### Appendix 1: The theoretical validity of responses, consistency test and regression analysis

#### The theoretical validity of responses and consistency test

The theoretical validity of responses was checked by testing the sign of attribute coefficients. It was hypothesised that respondents would prefer: a mixed level of practical activities (negative signs for the β1 to β3, see analytical plan below); peer support as well as the chance for people to use their existing skills and learn new ones but not a relaxing atmosphere (positive signs for β4, β5 and negative for β6); equally valued social opportunities (negative signs for β7 to β9); multiple times per week (negative signs for β10); drop at any time on available days (negative signs for β11 to β13); less costly options (negative sign for β14). Considering the average older age of the cohort of CM attendees, we expected carers would highly value their current experience (negative signs for the alternative specific constants, ASCs).

A dominant choice set (in which one service contains all preferred attribute levels and the other contains the least preferable levels) was added to check for consistency (Ryan et al. 2005).

#### Regression analysis and use of the data

For the DCE data, we utilised a Multinomial Logit (MNL) framework, following the guidelines outlined by McFadden (1973). We estimated a random effect utility function aligned with the principles of Random Utility Theory. According to this theory, an individual’s utility value assigned to an attribute within a choice scenario can be represented by a composite of two components: an explainable (fixed) component and an unexplainable (random) component.

A conditional logit was first fitted to the data, which provides a useful starting point in analysing choice data. To account for heterogeneity in preferences, we employed mixed logit models, testing different models with a fixed cost coefficient and also where we assumed to be random and log-normally distributed. A comparison of model fit based on the log-likelihood, Akaike information criterion (AIC) and Bayesian information criterion (BIC) indicated that the logit model fits the data better than the mixed logit models. More details on the logit models are available from the authors upon request. For simplicity, here we describe the conditional logit model presented in the paper, where the utility of choosing alternatives online U_online_ or in-person U_in-person_ (compared with ‘My MC’) can be specified as:

U_online_ = ASC_online_+ β1⋅ PRACTICALinfo+ β2⋅ PRACTICALphysical + β3⋅ PRACTICALbrain + β4⋅ EMOTIONALpeers- support + β5⋅ EMOTIONALlearning + β6⋅ EMOTIONALrelaxation + β7⋅ SOCIALactivities-others + β8⋅ SOCIALcommunity + β9⋅ SOCIALfriends + β10⋅ FREQUENCYmultiple + β11⋅ AVAILABILITYdropin-range + β12⋅ AVAILABILITYspecific + β13⋅ AVAILABILITYpre-appointed + β14⋅ COST + εi

U_in-person_ = ASC_in-person_+ β1⋅ PRACTICALinfo+ β2⋅ PRACTICALphysical + β3⋅ PRACTICALbrain + β4⋅ EMOTIONALpeers- support + β5⋅ EMOTIONALlearning + β6⋅ EMOTIONALrelaxation + β7⋅ SOCIALactivities-others + β8⋅ SOCIALcommunity + β9⋅ SOCIALfriends + β10⋅ FREQUENCYmultiple + β11⋅ AVAILABILITYdropin-range + β12⋅ AVAILABILITYspecific + β13⋅ AVAILABILITYpre-appointed + β14⋅ COST + εi

The choice of corresponding alternatives (1 = chosen, 0 = not chosen) was a dependent variable in the regression, and the attributes were independent variables. The ASC is an alternative specific constant capturing the variation in choices that is not explained by the attributes.

##### The direction of preferences and importance of characteristics

Our analysis focused on the direction of preferences within the Meeting Centre (MC) characteristics. A positive coefficient signifies that an increase in the variable corresponds to an increased likelihood of choosing the associated option. This insight provides a clear understanding of the factors that positively influence participants’ choices within the MC framework.

To discern the importance of various MC characteristics, we delved into the *p*value linked to each coefficient. A low *p*value, typically below 0.10, indicates the statistical significance of the variable’s impact on choices. Additionally, we considered the size of the coefficients; larger coefficients denote a more substantial influence on choices, highlighting the relative importance of specific MC attributes compared to those with smaller coefficients.

##### Ranking different MC configurations

We further use the MNL regression results to estimate utility scores obtained from given combinations of attribute levels and overall preference for specific MC configurations. From this we can learn about the relative ranking of alternative MC configurations by overall utility and trade-offs between attributes that may inform how MC services can be reconfigured to yield greater carer utility.

##### Willingness to pay

The marginal rate of substitution (MRS) between price and other (statistically significant) attributes were analysed. The MRS represents the maximum amount of money the respondent was willing to pay for one level of an attribute over another (see below). The calculation of the confidence interval of WTP adopted the delta method (Bliemer & Rose, 2013).

Marginal WTP = −𝛽*k*/𝛽14, where *k* = 1 to 13

##### Explicative factors for WTP

Interactions between the ASC and socio-economic and health-related characteristics were also conducted to explore the differences in preferences among different populations. Significance levels of 0.10, 0.05, and 0.001 indicate the probability threshold below which the observed results are considered statistically significant. For example, a significance level of 0.05 means that there is a 5% chance that the observed results occurred due to random variation alone, while a significance level of 0.001 indicates an even lower probability of 0.1%. Results are presented in Appendix 3.

## Appendix 2: Ranking for the 5 most common MCs currently available

**Table:**

Practical activities and support	Emotional support	Social opportunities	How often can you attend?	How available is it?	Cost per visit	Utility	Ranking
A mix of brain-stimulating and physical activities, and useful info	A mix of relaxation, using/learning skills and peer support	A mix of chatting with friends, meeting new people and doing activities	Multiple times a week	You can go whenever you want	About £15	−0.35	1
A mix of brain-stimulating and physical activities, and useful info	A mix of relaxation, using/learning skills and peer support	A mix of chatting with friends, meeting new people and doing activities	No more than once a week	You can go whenever you want	About £15	−0.65	2
A mix of brain-stimulating and physical activities, and useful info	A mix of relaxation, using/learning skills and peer support	A mix of chatting with friends, meeting new people and doing activities	No more than once a week	Drop in/connect online on a specific day	About £5	−0.75	3
A mix of brain-stimulating and physical activities, and useful info	A mix of relaxation, using/learning skills and peer support	A mix of chatting with friends, meeting new people and doing activities	No more than once a week	Drop in/connect online on a specific day	About £10	−0.97	4
A mix of brain-stimulating and physical activities, and useful info	A mix of relaxation, using/learning skills and peer support	A mix of chatting with friends, meeting new people and doing activities	Multiple times a week	Drop in/connect online on a range of days	About £15	−1.18	5

## Appendix 3. Explicative factors for marginal WTP

**Table:**

Explicative factors		WTP In-person Meeting Centre^a^	WTP St. deviation	Significance	Comments
Attendance with:	The carer attends the Meeting Centre together with the person they support	−17.12	2.05	10%	*Notably, respondents reported lower marginal WTP for the ‘My MC’ option when attending together with the person they support*. This finding may be influenced by the fact that Welsh MCs, as well as many Scottish MCs, which had good response rates to the questionnaire, tend to have lower costs for members but high carer attendance and engagement. Also, we may argue that there has been a shift in perceptions of what a MC entails. Originally, MCs were seen as places that catered to the needs of both members and family carers in a safe and enjoyable manner, where active support was provided to both individuals with dementia and their family carers. However, the DCE findings suggest that MCs are now increasingly viewed as day-care or respite places primarily for carers. Depending on this perspective, the expectations regarding costs and carer engagement may differ. If seen as a formal service, higher costs may be expected with lower carer engagement. On the other hand, if viewed as an informal social club, lower costs may be anticipated with higher carer engagement.
	The carer attends the Meeting Centre but the person they support is no longer able to attend	29.59	3.55	Not significant	N/A
	The carer attends the Meeting Centre as a former carer	67.52	8.10	5%	*The study found that carers who attend the MC as former carers expressed a preference for shifting from the ‘My MC’ option to an alternative in-person MC*. As former carers, individuals may have already fulfilled their caregiving responsibilities, either due to the passing of the person they cared for or a transition in care arrangements. In this new phase, they may seek a different type of support or interaction, and an alternative in-person MC could better cater to their evolving needs.
Age (carer)		−2.65	0.32	10%	*Additionally, the study found that younger carers exhibited lower marginal WTP for the ‘My MC’ option, or higher marginal WTP for a shift to an alternative option if they were caring for their parents*. This suggests that younger carers are more discerning in how they allocate their financial resources, potentially perceiving MCs more as a service and having less of a ‘status quo bias’ compared to older individuals. It is worth noting that older carers, particularly spousal carers, are more likely to live with the person with dementia, experience social isolation, and have their own health issues. Consequently, they may feel a greater need for support and have fewer alternatives, leading to less selectivity in their choices.
Gender Male (carer)		−38.36	4.60	1%	*The study findings indicate that male carers demonstrated a higher willingness to pay (WTP) for the ‘My MC’ option compared to female carers*. Men are less likely to attend MC, but, when they start, they love it and do not want to change. Women are more likely to have a caring role with children and other people to care for. They are more flexible and with experience of social groups. Societal norms and gender roles may influence the way male and female carers perceive their caregiving responsibilities. Male carers may have different expectations and preferences when it comes to the support and services they seek for themselves and the person they care for. This could result in a higher valuation of the attributes associated with the ‘My MC’ option. Male carers may have different financial resources and decision-making power compared to female carers. This could impact their willingness to allocate financial resources towards the ‘My MC’ option. Factors such as income levels, control over financial matters, and individual financial priorities might contribute to the observed difference in WTP.
Relationship to the person they support	Spouse or partner	−11.94	1.43	Not significant	N/A
	Son or daughter	15.37	1.84	10%	*The study found that carers who provide support to their parents expressed a willingness to pay for a shift from the ‘My MC’ option to an alternative in-person MC*. Caring for parents can present unique challenges and responsibilities. Carers supporting their parents may require specific types of support and services that are better addressed in an alternative in-person MC. These centres may offer tailored programs, activities, or resources that cater to the specific needs of older individuals or those caring for elderly parents.
	Other family member	−1.51	0.18	Not significant	N/A
	Friend	−100.80	12.10	Not significant	N/A
Age (person they support)		−3.64	0.44	5%	*The study revealed that carers supporting an older person expressed a lower WTP for the ‘My MC’ option compared to those caring for a younger person*. Carers supporting older individuals may face different financial circumstances compared to those caring for younger individuals. They may have additional financial responsibilities related to the care and support of older persons, such as healthcare expenses or other costs associated with aging. These financial constraints could influence their lower WTP for the MC option. Also, carers supporting older individuals may perceive that the MC offers fewer benefits or a lower level of impact for the older person compared to younger individuals. They might believe that the older person may have fewer opportunities (or less mobility) to actively engage in the activities or programs provided by the Meeting Centre, leading to a lower valuation of the service.
Gender Male (person they support)		18.40	2.21	5%	*The study found that carers supporting a male person, such as their husband or father, expressed a preference for a shift to an alternative MC*. Carers supporting male individuals may perceive that an alternative Meeting Centre better meets the specific needs and preferences of their male loved ones. They may believe that the alternative option offers activities, resources, or support that are more tailored to the interests and requirements of male participants (maybe choosier than their mother).
Health status (person they support)		2.69	0.32	Not significant	N/A

^a^Compared with My Meeting Centre.
